# A simulation and experiential learning intervention for labor and delivery providers to address HIV stigma during childbirth in Tanzania: study protocol for the evaluation of the MAMA intervention

**DOI:** 10.1186/s12884-023-05482-z

**Published:** 2023-03-16

**Authors:** Melissa H. Watt, Linda M. Minja, Mariam Barabara, Pendo Mlay, Maya J. Stephens, Gaudensia Olomi, Janeth Mlay, Virginie Marchand, Blandina T. Mmbaga, Olivia R. Hanson, Susanna R. Cohen

**Affiliations:** 1 Intermountain Healthcare, Department of Population Health Sciences, Spencer Fox Eccles School of Medicine, University of Utah, 295 Chipeta Way, Salt Lake, UT 84102 USA; 2 Kilimanjaro Clinical Research Institute, Moshi, Tanzania; 3 Department of Obstetrics and Gynecology, Kilimanjaro Christian Medical Center, Moshi, Tanzania; 4 Kilimanjaro Regional Department of Health, Moshi, Tanzania; 5 School of Medicine, Duke University, Durham NC, USA; 6 Department of Obstetrics and Gynecology, University of Utah, Salt Lake City, UT USA

**Keywords:** Tanzania, HIV, Stigma, Respectful maternity care, Simulation Training, Intervention

## Abstract

**Background:**

The experience of HIV stigma during intrapartum care can impact women’s trust in the health care system and undermine their long-term commitment to HIV care engagement. Delivery of respectful maternity care (RMC) to women living with HIV (WLHIV) can improve quality of life and clinical outcomes. The goal of this study is to conduct an evaluation of *MAMA* (Mradi wa Afya ya Mama Mzazi, Project to Support the Health of Women Giving Birth), a simulation team-training curriculum for labor and delivery providers that addresses providers’ instrumental and attitudinal stigma toward WLHIV and promotes the delivery of evidence-based RMC for WLHIV.

**Methods:**

The *MAMA* intervention will be evaluated among healthcare providers across six clinics in the Kilimanjaro Region of Tanzania. To evaluate the impact of MAMA, we will enroll WLHIV who give birth in the facilities before (n = 103 WLHIV) and after (n = 103 WLHIV) the intervention. We will examine differences in the primary outcome (perceptions of RMC) and secondary outcomes (postpartum HIV care engagement; perceptions of HIV stigma in the facility; internal HIV stigma; clinical outcomes and evidence-based practices) between women enrolled in the two time periods. Will also assess participating providers (n = 60) at baseline, immediate post, 1-month post training, and 2-month post training. We will examine longitudinal changes in the primary outcome (practices of RMC) and secondary outcomes (stigma toward WLHIV; self-efficacy in delivery intrapartum care). Quality assurance data will be collected to assess intervention feasibility and acceptability.

**Discussion:**

The implementation findings will be used to finalize the intervention for a train-the-trainer model that is scalable, and the outcomes data will be used to power a multi-site study to detect significant differences in HIV care engagement.

**Trial Registration:**

The trial is registered at clinicaltrials.gov, NCT05271903.

**Supplementary Information:**

The online version contains supplementary material available at 10.1186/s12884-023-05482-z.

## Background

HIV stigma during the intrapartum period (labor & delivery) can impact the birth experience of women living with HIV (WLHIV), and, in turn, can influence women’s long-term commitment to HIV care. Prevention of mother-to-child transmission (PMTCT) programs in Africa have achieved near-universal HIV testing for pregnant women and reduced infant HIV infections; however, overall retention in HIV care, especially during the postpartum period, has been suboptimal [1–5]. Our systematic review of PMTCT programs across Africa found that 27% of postpartum WLHIV were no longer in care at six months, rates substantially lower than other groups of people living with HIV [5]. Multiple studies have noted the impact of HIV-related stigma on care linkage and retention in PMTCT programs [4, 6–8]. Although data is limited, there is evidence that labor and delivery (L&D) providers may deliver suboptimal and stigmatizing care to WLHIV. Despite this, HIV stigma reduction interventions have not targeted the intrapartum period. In a review of interventions addressing stigma among healthcare providers, none were found to focus on L&D providers [9, 10]. Lack of engagement with L&D providers is a missed opportunity to change the trajectory of care for WLHIV.

Data from health facilities around the globe suggest that mistreatment and neglect are common during childbirth, which not only undermines women’s dignity but also contributes to poor maternal and child health outcomes [11, 12]. The World Health Organization has proposed a framework of respectful maternity care (RMC), which includes principles of dignity, privacy, confidentiality, freedom from harm, and informed choice [13]. RMC not only improves maternal and child health outcomes, but also reduces experiences of stigma, disrespect and abuse, which results in greater engagement and trust in the healthcare system [14, 15].

When HIV stigma is present during the intrapartum period, it undermines the delivery of RMC [16]. HIV stigma manifests both in the delivery of care and in the receipt of care by WLHIV. Stigma on the part of healthcare providers includes both *instrumental stigma* (avoidance and neglect, often driven by fear of occupational exposure) and *attitudinal stigma* (blame and judgment of people living with HIV) [17]. L&D providers who deliver respectful and intentionally non-stigmatizing care to WLHIV can help women to overcome both *internalized HIV stigma* (how they feel about themselves as a WLHIV) and *anticipated HIV stigma* (how they expect others will treat them). A respectful birth can also deepen women’s trust in the health care system, and motivate them to remain in HIV care during the postpartum period [18]. While this is important for all women, it is essential for WLHIV, for whom postpartum care engagement is necessary to ensure long-term health outcomes, including newborn HIV testing and long-term adherence to antiretroviral therapy (ART).

Healthcare providers are not immune to the cognitive biases that are deeply embedded in societal norms, and these biases directly impact the delivery of evidence-based practices for stigmatized populations [19]. Providers’ unconscious attitudes or implicit bias can manifest as subtle in isolation, yet the cumulative effects create mistrust between patients and providers, erode patients’ faith in the health care system, and significantly impede positive health outcomes [20–22].

Simulation training is a team-based experiential learning approach that helps providers refine clinical skills and improve inter-personal competencies, such as patient-provider communication. It integrates cognitive, technical, and behavioral objectives, building on shared experiences, and facilitating reflection and debriefing. Simulation learning can change providers’ attitudes and behaviors and improve the delivery of RMC [9]. Simulation training has the potential to reduce *instrumental stigma* by helping L&D providers develop the skills and self-efficacy to manage routine births and obstetric emergencies in WLHIV while protecting themselves from occupational exposure. It can also reduce *attitudinal stigma* by helping L&D providers reflect on their implicit biases and how these might be expressed and received by WLHIV.

The goal of this study is to conduct an evaluation of *MAMA* (*Mradi wa Afya ya Mama Mzazi*, Project to Support the Health of Women Giving Birth), a simulation team-training curriculum for L&D providers that addresses providers’ instrumental and attitudinal stigma toward WLHIV and promotes the delivery of evidence-based, respectful maternity care for WLHIV.

## Methods

### Study overview

**Fig. 1:**
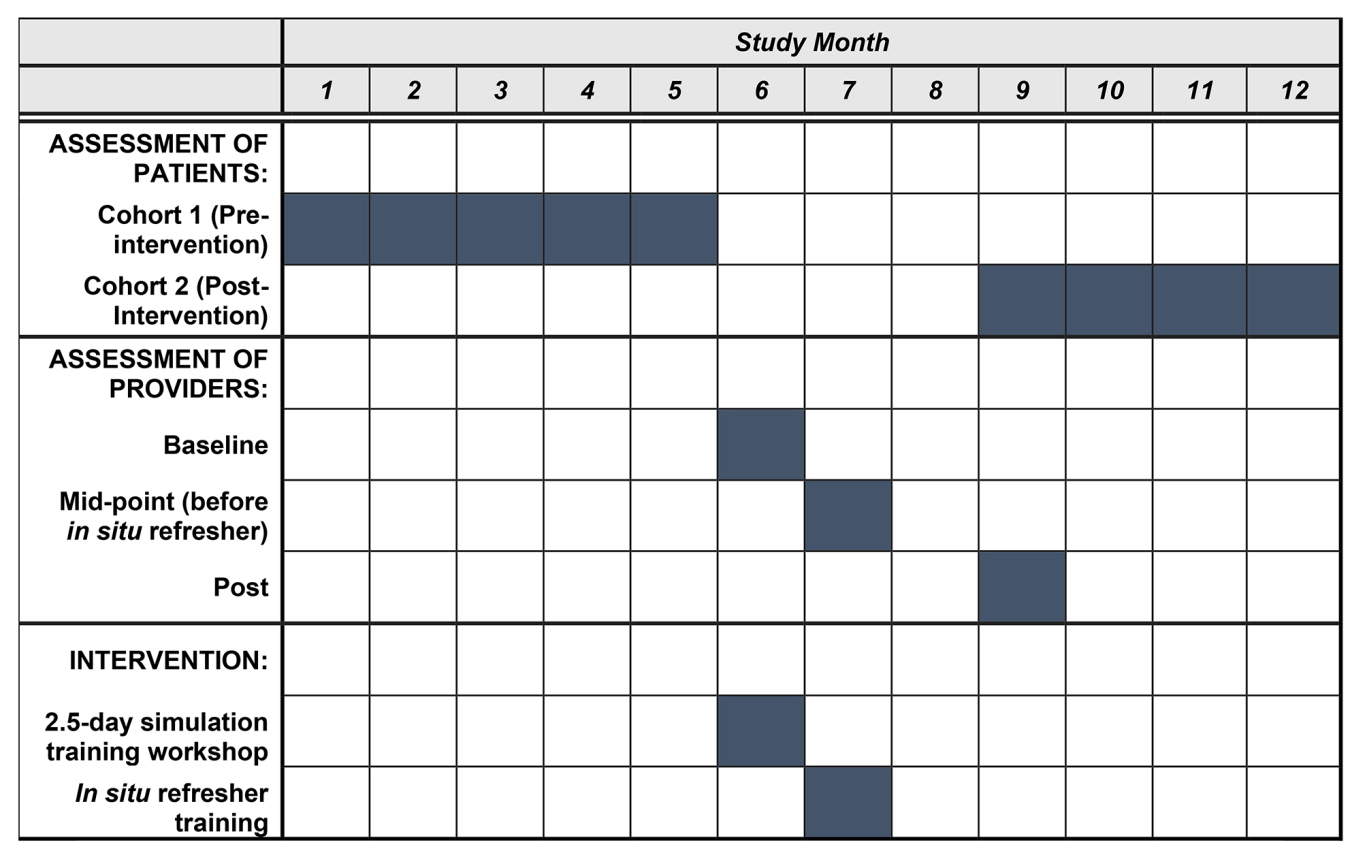
Study Timeline

This study will evaluate the *MAMA* training intervention among healthcare providers across six clinics in the Kilimanjaro Region in northern Tanzania. The study will evaluate the impact of the intervention on patient outcomes using a quasi-experimental pre-post design (comparing patient-reported outcomes in the pre- and post-intervention periods), and the impact on provider outcomes using pre- and post-test assessment. The collection of robust quality assurance data will support the refinement of the intervention and prepare us to implement a future clinic-randomized trial. Table 1 summarizes key elements of the study, and Fig. 1 provides a timeline of the key study elements.

**Table 1:** Study summary (Template adapted from the World Health Organization Trial Registration Data Set)

DATA CATEGORY	INFORMATION
**Title**	Pilot evaluation of MAMA (*Mradi wa Afya ya Mama Mzazi*, Project to Support the Health of Women Giving Birth): A simulation and experiential learning intervention for labor and delivery providers to address HIV stigma during childbirth in Tanzania
**Primary registry and trial identifying number**	ClinicalTrials.gov NCT05271903 Registered March 8, 2022
**Secondary identifying numbers**	R21TW012001 (U.S. NIH Grant/Contract)
**Primary funder**	Fogarty International Center (NIH)
**Contact for public queries**	Melissa Watt, PhD melissa.watt@hsc.utah.edu
**Countries of recruitment**	Tanzania
**Health condition(s) or problem(s) studied**	Health services research
**Key inclusion criteria**	Birthing women: Over age 18 Gave birth in a study clinic Diagnosed with HIV Labor and delivery providers: Over age 18 Employed by a study clinic Works clinically in the labor and delivery ward
**Study type**	Interventional (Clinical Trial) Allocation: N/A Intervention model: Single group assignment
**Date of first enrollment**	Birthing women: March 14, 2022 Labor and delivery providers: November 15, 2022 [anticipated]
**Target sample size**	Birthing women: 206 Labor and delivery providers: 60
**Recruitment status**	Recruiting
**Primary outcome(s)**	Birthing women: Perceptions of respectful maternity care Labor and delivery providers: Practices of respectful maternity care
**Key secondary outcomes**	Birthing women: Postpartum HIV care engagement Perceptions of HIV stigma in the facility Internalized HIV stigma Clinical outcomes and evidence-based birth practices Labor and delivery providers: Stigma toward WLHIV Self-efficacy in delivering intrapartum care

### Ethical approval and registration

The study has been approved by the ethical review committees at the University of Utah (Protocol 00143918), Kilimanjaro Christian Medical Center (Protocol 2056), and National Institute for Medical Research in Tanzania (Protocol 3853). The trial is registered at clinicaltrials.gov (NCT05271903).

### Trial status

This trial was registered at ClinicalTrials.gov on 8 March 2022 (NCT05271903). The first patient participant was enrolled on 14 March 2022. We anticipate that the first provider participant will be enrolled in November 2022. Participant recruitment and enrollment is ongoing and expected to be completed by June 2023, with final follow-up expected by August 2023.

### Study setting

The study will be conducted in six primary health centers, including 4 government hospitals and 2 Designated District Hospitals (DDH), which are owned and operated by a faith-based organization and supported by the government. The study will be located in Moshi (urban) and Rombo (rural), both in the Kilimanjaro Region. In 2019, the six clinics together provided intrapartum services for 258 WLHIV.

### Participants

The study will enroll approximately 400 birthing women and 60 labor and delivery providers. Birthing women will be eligible if they delivered a baby in a study facility during either the pre-intervention enrollment period or post-intervention enrollment period. Given our primary focus on WLHIV, we will over-sample this population, with a goal of 103 WLHIV recruited in each period. For each WLHIV enrolled in the study, we will recruit up to two women who are not living with HIV who gave birth the same day, in the same facility, and are similar in age (+/- 5 years) and parity (nulliparous, primiparous, or multiparous). Providers will be eligible if their clinical duties include attending and supporting childbirth in any of the six study facilities.

### Screening and recruitment

#### Birthing women

Participants will be recruited from the maternity wards of the six participating facilities. When a WLHIV is in the postpartum ward following childbirth, a member of the clinic staff will introduce the study; if the woman is interested, the facility staff will send a text message to the study coordinator about a potential participant. The study staff will meet with the woman to introduce the study and answer questions. The informed consent process will be conducted orally; to ensure that HIV status is not involuntarily disclosed to others, the consent form does not include any information about HIV status. The same process is repeated with HIV-negative women who meet eligibility criteria.

#### Providers

Facility leadership will help to identify facility providers who can be released from duty to attend the two and a half day MAMA training. Research staff will meet with providers as a group to describe research activities, including the time commitment. When providers report to the training, they will be given the informed consent form to review; prior to signing, they will have an opportunity to ask questions.

### Procedures

#### Birthing women

After providing consent, patient participants will complete a structured survey using audio computer-assisted self-interview (ACASI) technology on tablets running Questionnaire Development System (QDS) software. The ACASI modality ensures patient privacy and minimizes social desirability bias to improve data validity [23]. Participants will complete the assessment on individual tablets where they can read the screen and listen to recorded audio for the questions and response options in Kiswahili. The survey will take approximately 30–45 min to complete. Research staff will be present in the room to aid participants if needed. The study staff will review the patient’s medical records to collect clinical information about the birth (e.g., patient history, clinical management, complications, birth outcomes). A locator form will be completed to document information about how the participant can best be reached for a brief follow-up survey. Approximately three weeks after the birth, the research staff will call the participant to administer a brief survey focused on the health and well-being of the woman and her child, and engagement in post-partum clinical care. The researcher will attempt follow-up for a two week period before identifying a participant as lost to follow up. The follow-up survey will take approximately 10–15 min and take place over the phone.

#### Providers

After providing informed consent, providers will self-complete a paper-based survey evaluating RMC practices, stigma towards WLHIV, and self-efficacy and clinical knowledge of obstetric emergency care. The providers will then participate in the *MAMA* intervention, which is a two-and-a-half day workshop in a central location, followed by a one-day in situ refresher training 1–2 months later at each of the 6 facilities. Follow-up surveys will be conducted at three time points: immediate post training, pre in situ refresher, and two months post in situ refresher. The research team will make an appointment for the follow-up surveys by phone, and then visit the participant on site during a working shift in order to administer the survey. If the participant has been transferred to a new clinical site within the Kilimanjaro Region, they will visit the new site.

#### Data management

For the patient ACASI data collection, data files will be transferred and uploaded daily by the local data manager in Tanzania. The files will be stored on a secured drive (Box.com) and files will be reviewed weekly for quality assurance. For the provider data collection, paper surveys will be entered into a RedCap database. Limited access to stored data, only identifiable by a unique ID, will be provided to authorized research staff.

### Intervention condition

The *MAMA* intervention is a two-and-a-half day simulation training workshop for labor and delivery providers, followed by a one-day in situ refresher course 1–2 months later. The *MAMA* intervention is based on PRONTO International’s simulation training program to improve obstetric care. PRONTO International is a non-governmental organization whose simulation and team-training curriculum has been implemented in a variety of clinical settings, from primary health centers to district and regional hospitals [24–27]. The PRONTO model has been applied and evaluated in multiple settings, including East Africa, and has been shown to improve provider skills [25, 27, 28], reduce morbidity and mortality [26, 29–31], and improve the use of RMC practices [9]. The interactive curriculum is based on simulation and debriefing of clinical scenarios, case-based learning, skills stations, and teamwork activities.

Adaptation of the PRONTO model to create the MAMA intervention followed the ADAPT-ITT model [32]. In order to understand the experiences of RMC for WLHIV, we conducted qualitative in-depth interviews with pregnant and postpartum women (n = 36), focus group discussions with L&D providers in the study clinics (n = 6) and focus group discussions with nurse-midwifery students (n = 2). This data allowed us to best target the key actionable drivers of HIV stigma among health care providers, and to address the clinical needs of the provider population. The curriculum was drafted by the inter-disciplinary international study team (including nurse midwives, an obstetrician, a pediatrician, and a behavioral scientist), and in consultation with the PRONTO leadership. We received two rounds of input from a stakeholder advisory board, which included representation from the municipal and regional Departments of Health, HIV advocates, maternal and child health advocates, community representative and nurse and midwife educators and experts. We conducted “theater testing” [33] of the simulation models in Tanzania, in order to refine the training materials.

The final two and a half -day training curriculum includes three simulation scenarios, knowledge reviews (interactive case-based learning activities), teamwork and communication activities, and interactive activities that address bias and stigma. Activities are intentionally ordered so that the learning is scaffolded and the opportunities and challenges require increasing integration and application of new skills and knowledge on clinical practices, bias, and RMC. The in-situ follow-up training includes a structured review of training materials, one simulation scenario, and flexibility to focus on areas the trainers identify as needing additional work. The in-situ training will highlight facility readiness and systems level weaknesses that can be addressed by the participating team.

The training curriculum will be delivered by four PRONTO Master Trainers who are based in Kenya. The 2-day training will be offered twice (with approximately 30 providers at each training). The training will be hosted at the KCMC Hospital, which is the tertiary referral hospital for all the study facilities.

### Outcomes

#### Primary outcome for birthing women

##### Perceptions of respectful maternity care

will be measured with a 31-item measure of person-centered maternity care developed by Afulani and colleagues [34, 35]. The measure asks a woman to report on the quality of care she received as it relates to domains of RMC. Items will be summed and standardized on a scale of 0-100. The scale includes three sub-scales: dignity and respect (6 items), communication and autonomy (9 items), and supportive care (15 items).

#### Secondary outcomes for birthing women

##### Postpartum HIV care engagement

will be measured in the phone follow-up interview after participants have been discharged from the hospital. Participants will be asked a single question (yes/no) about whether they have attended any postpartum visits to the prevention of mother to child transmission (PMTCT) clinic. Participants will also be asked questions about adherence to antiretroviral (ARV) medication in the prior four days. If participants have not attended any PMTCT visits in the postpartum period, or if they have missed any ARV medication in the prior four days, they will be considered to have poor care engagement.

##### Perceptions of HIV stigma in the facility

will be assessed among WLHIV using an 11-item measure that was adapted from two measures of HIV stigmatizing behaviors of health care workers [36, 37]. The measure asks women to rate their perception of stigma by health care providers (e.g., Do you feel like providers avoided touching you because of your HIV status? Did a provider say something negative to you about your HIV status?). Item responses are on a Likert scale (0–3), and will be summed for a possible range 0–33.

##### Internalized HIV stigma

will be assessed among WLHIV using Scale A of the HIV and Abuse Related Shame Inventory (HARSI) [38]. The measure includes 13 items that ask about experiences of internalized stigma (e.g., It is hard to tell other people about my HIV infection. I am ashamed that I have HIV.). Items are on a Likert scale (0–4), and will be summed for a possible range 0–52.

##### Clinical outcomes and evidence-based birth practices

will be assessed via medical record reviews and include mode of delivery (cesarean vs. vaginal), neonatal ART administration, completion of the 3 steps of Active Management of the Third Stage of Labor (AMTSL), use and timing of oxytocin, episiotomy, indication for cesarean section, and whether the patient was transferred in or out of the facility.

#### Primary outcome for providers

##### Practices of respectful maternity care

will be assessed using a measure of person-centered maternity care among providers, which was validated in Kenya and Ghana [39]. The 9-item measure asks providers how often they have done certain practices in the preceding one month (e.g., How often did you ask patients if they had any questions?). Items are on a Likert scale (0–3) and will be summed for a possible range 0–27.

#### Secondary outcomes for providers

##### Stigma toward WLHIV

will be assessed with three measures capturing: fear of HIV acquisition in providing care for WLHIV (9 items, e.g., How worried are you about getting HIV if you touched without gloves the skin of someone with HIV?); extra precautions in providing care for WLHIV (7 items, e.g., When taking care of a woman with HIV, how often do you wear double gloves?); and attitudes toward WLHIV (6 items, e.g., People living with HIV could have avoided HIV if they had wanted to). The measures of fear of HIV acquisition and extra precautions were both adapted from Nyblade’s work evaluating HIV stigma in a healthcare setting in Ghana [40], with additional items added specifically for the childbirth setting. The 6-item measure of stigmatizing attitudes towards WLHIV was developed by Nyblade based on field testing in six countries [37]. All items are measured on a 0–3 scale. A mean score will be calculated to assess overall stigma toward WLHIV, and separate domain scores will capture fear of HIV acquisition, extra precautions, and attitudes toward WLHIV.

##### Self-efficacy in delivering intrapartum care

will be assessed using measures that PRONTO has used in previous evaluations [10]. The measure includes nine items, and participants will be asked to rate their confidence in various clinical scenarios on a scale of 0-100 (e.g., How confident are you in handling a postpartum hemorrhage? How confident are you in talking to a woman and her family about her condition during an emergency?). Seven of the nine items will be repeated and asked specifically in the context of caring for WLHIV (e.g., How confident are you in handling a postpartum hemorrhage in a woman with HIV?). We will calculate mean scores (ranging from 0 to 100), separately for the general measure and for the measure specific to caring for WLHIV.

##### Provider burnout

will be assessed using a 2-item measure [41] that were adapted from the Maslach Burnout Inventory [42]. The first question defines burnout as chronic emotional exhaustion in one’s job, and asks how often the participant experiences this. The second question asks how often the participant has felt more callous toward people since they took their job. Both questions have response options on a scale of 1 (“Never”) to 7 (“Every Day”). Participants will be considered to have symptoms of burnout if they score a minimum of 4 (“a few times a month”) on either the first question (emotional exhaustion) or the second question (depersonalization). This measure will not be evaluated as an outcome, but rather will rather be used as a confounding factor in providers’ ability to provide RMC.

### Quality assurance (QA) data

In order to assess the implementation of *MAMA* and therefore its potential for scalability, we will focus on intervention feasibility and acceptability, as defined by Procter et al. [12]. Feasibility will be assessed based on the proportion of the eligible providers who attend the intervention, and the proportion of the total intervention time attended. Attendance rosters will track who attends and for how long, and the intervention will be considered feasible if at least 65% of the eligible participants attend a minimum of 75% of the intervention time. During the trainings, we will have an individual dedicated to taking detailed notes of participants’ discussion and input. These notes will be thematically coded to capture the quality and content of participation, and to identify areas to improve future iterations of the curriculum.

Acceptability will be assessed in the immediate post surveys with participating L&D providers. Participants will be asked to rate the usefulness of the intervention for their clinical practice and their satisfaction with the intervention format and facilitators. The intervention will be considered acceptable if the mean scores of items are greater than 4.0 (on a scale of 1 to 5). Participants will also be asked to provide open-ended feedback about their favorite and least favorite aspects of the intervention and their suggestions for modification and scale-up. Responses will be thematically coded and summarized.

### Statistical analysis

#### Primary outcome: women giving birth

We will first compare the RMC full scale scores between pre- and post- intervention periods. We will use ordinary least square regressions (OLS) to model RMC scores with time point (pre-intervention or post-intervention) as an indicator variable while controlling for demographic characteristics, clinical characteristics, and facility level factors. We will then repeat the analysis for the three RMC sub-scales (dignity and respect; communication and autonomy; supportive care). To assess whether HIV status moderates the impact of the intervention, an interaction term for HIV status and timepoint will be added in the OLS models. A significant interaction term will be evidence that HIV status moderated the effect of the intervention.

#### Secondary outcomes: women giving birth

The outcome measures of perceptions of HIV stigma in the facility and internalized HIV stigma will both be analyzed continuously. OLS models will used to examine differences in scores in the samples enrolled in the pre-intervention period and the post-intervention period. The outcomes of postpartum HIV care engagement, clinical outcomes, and presence of evidence-based maternity practices will be analyzed categorically. Chi square tests will be used to assess differences in the proportion of WLHIV who had the presence of characteristics (e.g., poor care engagement, cesarean delivery) in the pre-intervention period, compared with the post-intervention period.

#### Primary outcome: L&D providers

To assess changes in practices of RMC among providers, we will use a random mixed effect model. We will model RMC scores with timepoint (pre-intervention, immediate post-training, pre in situ refresher, and two months post in situ refresher) as a predictor variable.

#### Secondary outcomes: L&D providers

The outcome measures of stigma towards WLHIV and self-efficacy in providing intrapartum care will both be analyzed as continuous variables. We will following the same analytic strategy as we have outlined for the primary outcome, using a random mixed effect model and including timepoint as a predictor variable. As a follow-up analysis, we will assess whether providers’ reported burnout at baseline is associated with our primary and secondary outcome variables at baseline, and with change in the outcome variables over time.

### Sample size considerations

A power calculation was conducted *a priori*, considering our primary outcome of RMC among WLHIV. Power analysis showed that we will need 103 WLHIV at each time point to be able to detect a moderate intervention effect (d = 0.4) with two-sided significance level of 5% and 80% power, hence a total of 206. To allow comparison with HIV negative women, a similar sample size with some matching characteristics with WLHIV will be enrolled, but low response rate and some operational challenges are expected in his group; therefore, up to 200 HIV negative women will be invited to participate. The sample size for providers was based on convenience; we hope to reach at least 65% of all eligible providers in the six study facilities, which we estimate will provide a sample of 60 providers.

### Dissemination

A study advisory board has been established to provide ongoing stakeholder input on the study and share emerging data and findings. The board will be convened for three half-day workshops during the study: initially, for input on intervention content; mid-way, for feedback on the curriculum and preliminary findings; and at the conclusion, for interpretation/dissemination of results. Advisory board members will be updated on the study progress.

At the conclusion of the study, we will conduct a feedback forum with a larger audience of stakeholders. During the forum, the team will share the findings of the study and facilitate a discussion about the implications of the data for future research and practice. Results will also be published in peer-reviewed journals and presented at appropriate scientific meetings, including regional, national, and international meetings. Authorship eligibility guidelines will follow the authorship guidelines of the International Committee for Medical Journal Editors (www.icmje.org).

All study investigators, along with the data management team, will have access to the final trial dataset. Researchers from outside the team can request access; data can be shared with a data transfer agreement from the respective Institutional Review Boards and within the constraints required for the protection of confidentiality for study subjects.

### Study organization

As the principal investigators of the MAMA intervention, Drs. Melissa Watt, Susanna Cohen, and Blandina Mmbaga are charged with co-leading the study. They will ensure the completion and integrity of the study by managing and monitoring study activities and the reporting of study findings. They will facilitate collaboration between the University of Utah and KCMC by initiating and maintaining communication between these two institutions and the study staff at both locations. Drs. Watt, Cohen, and Mmbaga will monitor the ethical overall conduct of research activities and be responsible for overseeing compliance of financial expenditures in accordance with sponsoring agency regulations.

The faculty investigator and PhD candidate in the study, Dr. Mlay and Mrs. Barabara, will bring expertise on maternal and infant care delivery and HIV engagement to the MAMA intervention. They will support the scientific oversight of the study, meeting weekly with study staff and providing on-going supervision and support.

A minimum of one data collection staff member will be the point person for each of the clinical sites and be responsible for recruiting participants and obtaining study data through surveys (using ACASI technology) and qualitative interviews. The data management team, led by statistician Linda Minja at KCMC, will be responsible for storing, analyzing, and interpreting quantitative data. The team will clean data and code measures at each time point in order to ensure that the data is valid and easily interpreted.

To elicit stakeholder input, we have established a study advisory board (see *Dissemination* section) that includes representatives from the Tanzanian Ministry of Health, leadership in the study clinics, community-based organizations, and members from the KCMC HIV Community Advisory Board.

## Discussion

The MAMA intervention is a provider training that was designed to help providers develop interpersonal and clinical skills to support the births of WLHIV. The intervention is based on the PRONTO model of simulation training, which has shown significant impacts on clinical outcomes [25, 26, 28, 29] and RMC practices [9] in multiple countries. This is the first time that the model will be adapted to support the births of WLHIV. In this study, we will deliver the MAMA intervention to 60 providers and assess outcomes in WLHIV (comparing patient-reported and clinical outcomes in WLHIV in the periods before and after the training), as well as outcomes in participating providers (comparing knowledge, attitudes and practices before and after the intervention). The implementation findings will be used to finalize the intervention for a train-the-trainer model that is scalable, and the outcomes data will be used to power a study to detect significant differences in HIV care engagement.

As we implement this study, we are aware of some key limitations of the study design. First, we do not have preliminary data to predict the mean and variance of our primary outcome; therefore, it is possible that our study is under-powered to detect significant differences. Second, the measurement of a key secondary outcome (HIV care engagement) is dependent on follow-up of patients following discharge from the labor ward. Given the time demands of women in the early postpartum period, and the possibility that some women may relocate to have additional support in caring for a new child, we may have challenges in retaining women for the follow-up survey. Similarly, we may face challenges in retaining providers, especially if they are reassigned to other clinical settings. Finally, the impact of the training on patient outcomes is dependent on a critical mass of providers receiving the training. Although we will offer the training twice and we will provide an honorarium for participating providers, we acknowledge that we may face challenges in getting the majority of providers trained in the MAMA intervention. Despite these limitations, this study will provide important data both to modify the intervention for future scalability and also to inform a future clinical trial.

In conclusion, WLHIV who are giving birth are a vulnerable population. While much work has been done to improve the antenatal care continuum for WLHIV through prevention of mother to child transmission programs, the clinical juncture of childbirth for WLHIV has received scant attention [43]. This study will fill an important gap by building the capacity and skills of L&D providers to provide evidence-based, respectful intrapartum care to WLHIV. If successful, the MAMA intervention can be scaled-up to be part of the training curriculum for midwifery students and other L&D providers.

## Electronic supplementary material

Below is the link to the electronic supplementary material.

**Supplementary material 1**

## Data Availability

Not applicable.

## Declarations

### Protocol version

The original protocol (Version 1) was approved by the ethical review committees in August 2021. The published protocol is current as of August 24, 2022. Protocol modifications will be submitted to the relevant ethical review committees and modified on clinicaltrials.gov.

## Abbreviations

ACASI: Audio computer-assisted self-interview
AMTSL: Active Management of Third Stage of Labor
ART: Antiretroviral therapy
DDH: Designated District Hospital
FGD: Focus group discussions
HIV: Human immunodeficiency syndrome
IDI: In-depth interviews
L&D: Labor and delivery
MAMA: Mradi wa Afya ya Mama Mzazi, Project to Support the Health of Women Giving Birth
OB/GYN: Obstetrics and Gynecology
OLS: Ordinary least square
PMTCT: Prevention of Mother to Child Transmissions
RCT: Randomized control trial
RMC: Respectful maternity care
WLHIV: Women Living with HIV

## References

[1] Turan JM, Miller S, Bukusi EA, Sande J, Cohen CR. HIV/AIDS and maternity care in Kenya: how fears of stigma and discrimination affect uptake and provision of labor and delivery services. AIDS Care. 2008;20(8):938–45. 10.1080/09540120701767224 PMC6788285 18777222

[2] Sando D, Kendall T, Lyatuu G, Ratcliffe H, McDonald K, Mwanyika-Sando M, et al. Disrespect and Abuse During Childbirth in Tanzania: Are Women Living With HIV More Vulnerable? J Acquir Immune Defic Syndr 1999. 2014;67(Suppl 4):S228–34. 10.1097/QAI.0000000000000378 PMC4251905 25436822

[3] Cichowitz C, Watt MH, Mmbaga BT. Childbirth experiences of women living with HIV: a neglected event in the prevention of mother-to-child transmission care continuum. AIDS. 2018;32(11):1537–9. 10.1097/QAD.0000000000001860 PMC6029874 29762160

[4] Watt MH, Cichowitz C, Kisigo G, Minja L, Knettel BA, Knippler ET, et al. Predictors of postpartum HIV care engagement for women enrolled in prevention of mother-to-child transmission (PMTCT) programs in Tanzania. AIDS Care. 2019;3(6):687–98. 10.1080/09540121.2018.1550248 PMC6443456 30466304

[5] Knettel BA, Cichowitz C, Ngocho JS, Knippler ET, Chumba LN, Mmbaga BT, et al. Retention in HIV Care during pregnancy and the Postpartum Period in the option B + era: systematic review and Meta-analysis of studies in Africa. JAIDS J Acquir Immune Defic Syndr. 2018;15(5):427–38. 10.1097/QAI.0000000000001616 PMC5844830 29287029

[6] Gesesew HA, Tesfay Gebremedhin A, Demissie TD, Kerie MW, Sudhakar M, Mwanri L. Significant association between perceived HIV related stigma and late presentation for HIV/AIDS care in low and middle-income countries: A systematic review and meta-analysis. Paraskevis D, editor. PLOS ONE. 2017;12(3):e0173928. 10.1371/journal.pone.0173928 PMC5373570 28358828

[7] Buregyeya E, Naigino R, Mukose A, Makumbi F, Esiru G, Arinaitwe J, et al. Facilitators and barriers to uptake and adherence to lifelong antiretroviral therapy among HIV infected pregnant women in Uganda: a qualitative study. BMC Pregnancy Childbirth. 2017;17(1):94. 10.1186/s12884-017-1276-x PMC5360052 28320347

[8] McMahon SA, Kennedy CE, Winch PJ, Kombe M, Killewo J, Kilewo C, Stigma. Facility constraints, and personal disbelief: why women disengage from HIV Care during and after pregnancy in Morogoro Region, Tanzania. AIDS Behav. 2017;21(1):317–29. 10.1007/s10461-016-1505-8 PMC5218979 27535755

[9] Afulani PA, Aborigo RA, Walker D, Moyer CA, Cohen S, Williams J. Can an integrated obstetric emergency simulation training improve respectful maternity care? Results from a pilot study in Ghana. Birth. 2019;46(3):523–32. 10.1111/birt.12418 30680785

[10] Afulani PA, Dyer J, Calkins K, Aborigo RA, Mcnally B, Cohen SR. Provider knowledge and perceptions following an integrated simulation training on emergency obstetric and neonatal care and respectful maternity care: a mixed-methods study in Ghana. Midwifery. 2020;85:102667. 10.1016/j.midw.2020.102667 32114318

[11] Bohren MA, Vogel JP, Hunter EC, Lutsiv O, Makh SK, Souza JP, et al. The Mistreatment of Women during Childbirth in Health Facilities Globally: A Mixed-Methods Systematic Review. Jewkes R, editor. PLOS Med. 2015;12(6):e1001847. 10.1371/journal.pmed.1001847 PMC4488322 26126110

[12] Bohren MA, Mehrtash H, Fawole B, Maung TM, Balde MD, Maya E, et al. How women are treated during facility-based childbirth in four countries: a cross-sectional study with labour observations and community-based surveys. The Lancet. 2019;394(10210):1750–63. 10.1016/S0140-6736(19)31992-0 PMC6853169 31604660

[13] Oladapo O, Tunçalp Ö, Bonet M, Lawrie T, Portela A, Downe S, et al. WHO model of intrapartum care for a positive childbirth experience: transforming care of women and babies for improved health and wellbeing. BJOG Int J Obstet Gynaecol. 2018;125(8):918–22. 10.1111/1471-0528.15237 PMC6033015 29637727

[14] Rathert C, Wyrwich MD, Boren SA. Patient-centered care and outcomes: a systematic review of the literature. Med Care Res Rev. 2013;70(4):351–79. 10.1177/1077558712465774 23169897

[15] Rubashkin N, Warnock R, Diamond-Smith N. A systematic review of person-centered care interventions to improve quality of facility-based delivery. Reprod Health. 2018;15(1):169. 10.1186/s12978-018-0588-2 PMC6180507 30305129

[16] World Health Organization. WHO recommendations on intrapartum care for a positive childbirth experience [Internet]. WHO. 2018. Available from: https://extranet.who.int/rhl/guidelines/who-recommendations-intrapartum-care-positive-childbirth-experience 30070803

[17] Nyblade L, Stockton MA, Giger K, Bond V, Ekstrand ML, Lean RM, et al. Stigma in health facilities: why it matters and how we can change it. BMC Med. 2019;17(1):25. 10.1186/s12916-019-1256-2 PMC6376713 30764806

[18] Hodgson I, Plummer ML, Konopka SN, Colvin CJ, Jonas E, Albertini J, et al. A Systematic Review of Individual and Contextual Factors Affecting ART Initiation, Adherence, and Retention for HIV-Infected Pregnant and Postpartum Women. Newell ML, editor. PLoS ONE. 2014;9(11):e111421. 10.1371/journal.pone.0111421 PMC4221025 25372479

[19] FitzGerald C, Hurst S. Implicit bias in healthcare professionals: a systematic review. BMC Med Ethics. 2017;18(1):19. 10.1186/s12910-017-0179-8 PMC5333436 28249596

[20] Chapman EN, Kaatz A, Carnes M. Physicians and Implicit Bias: how doctors may unwittingly perpetuate Health Care Disparities. J Gen Intern Med. 2013;28(11):1504–10. 10.1007/s11606-013-2441-1 PMC3797360 23576243

[21] Cruz D, Rodriguez Y, Mastropaolo C. Perceived microaggressions in health care: a measurement study. Zaller N. editor PLOS ONE. 2019;5(2):e0211620. 10.1371/journal.pone.0211620 PMC6363167 30721264

[22] Walls ML, Gonzalez J, Gladney T, Onello E. Unconscious Biases: Racial Microaggressions in American Indian Health Care. J Am Board Fam Med. 2015;28(2):231–9. 10.3122/jabfm.2015.02.140194 PMC4386281 25748764

[23] Watt MH, Knippler ET, Minja L, Kisigo G, Knettel BA, Ngocho JS et al. A counseling intervention to address HIV stigma at entry into antenatal care in Tanzania (Maisha): study protocol for a pilot randomized controlled trial. Trials [Internet]. 2019;20(1). [cited 2020 Feb 24] Available from: https://trialsjournal.biomedcentral.com/articles/10.1186/s13063-019-3933-z 10.1186/s13063-019-3933-z PMC6937735 31888700

[24] Fahey JO, Cohen SR, Holme F, Buttrick ES, Dettinger JC, Kestler E (2013). Promoting Cultural Humility during Labor and Birth: putting Theory into Action during PRONTO Obstetric and neonatal emergency training. J Perinat Neonatal Nurs.

[25] Walker DM, Holme F, Zelek ST, Olvera-García M, Montoya-Rodríguez A, Fritz J, et al. A process evaluation of PRONTO simulation training for obstetric and neonatal emergency response teams in Guatemala. BMC Med Educ. 2015;15(1):117. 10.1186/s12909-015-0401-7 PMC4513701 26206373

[26] Walker DM, Cohen SR, Fritz J, Olvera-García M, Zelek ST, Fahey JO, et al. Impact evaluation of PRONTO Mexico: A Simulation-Based program in Obstetric and neonatal Emergencies and Team Training. Simul Healthc J Soc Simul Healthc. 2016;11(1):1–9. 10.1097/SIH.0000000000000106 PMC5367503 26312613

[27] Dettinger JC, Kamau S, Calkins K, Cohen SR, Cranmer J, Kibore M, et al. Measuring movement towards improved emergency obstetric care in rural Kenya with implementation of the PRONTO simulation and team training program. Matern Child Nutr. 2018;14:e12465. 10.1111/mcn.12465 PMC6866198 29493898

[28] Vail B, Morgan MC, Spindler H, Christmas A, Cohen SR, Walker DM. The power of practice: simulation training improving the quality of neonatal resuscitation skills in Bihar, India. BMC Pediatr. 2018;18(1):291. 10.1186/s12887-018-1254-0 PMC6122678 30176831

[29] Walker D, Otieno P, Butrick E, Namazzi G, Achola K, Merai R, et al. Effect of a quality improvement package for intrapartum and immediate newborn care on fresh stillbirth and neonatal mortality among preterm and low-birthweight babies in Kenya and Uganda: a cluster-randomised facility-based trial. Lancet Glob Health. 2020;8(8):e1061–70. 10.1016/S2214-109X(20)30232-1 PMC7388203 32710862

[30] Kestler E, Ambrosio G, Hemming K, Hughes JP, Matute J, Moreno M, et al. An integrated approach to improve maternal and perinatal outcomes in rural Guatemala: A stepped-wedge cluster randomized trial. Int J Gynecol Obstet. 2020;ijgo.13262. 10.1002/ijgo.13262 32524605

[31] Ghosh R, Spindler H, Morgan MC, Cohen SR, Begum N, Gore A, et al. Diagnosis and management of postpartum hemorrhage and intrapartum asphyxia in a quality improvement initiative using nurse-mentoring and simulation in Bihar, India. Kamath-Rayne B, editor. PLOS ONE. 2019;14(7):e0216654. 10.1371/journal.pone.0216654 PMC6611567 31276503

[32] Wingood GM, DiClemente RJ. The ADAPT-ITT model: a novel method of adapting evidence-based HIV Interventions. J Acquir Immune Defic Syndr 1999. 2008;47 Suppl 1:S40-46. 10.1097/QAI.0b013e3181605df1 18301133

[33] Hegland PA, Aarlie H, Strømme H, Jamtvedt G. Simulation-based training for nurses: systematic review and meta-analysis. Nurse Educ Today. 2017;54:6–20. 10.1016/j.nedt.2017.04.004 28456053

[34] Afulani PA, Diamond-Smith N, Golub G, Sudhinaraset M. Development of a tool to measure person-centered maternity care in developing settings: validation in a rural and urban kenyan population. Reprod Health. 2017;14(1):118. 10.1186/s12978-017-0381-7 PMC5610540 28938885

[35] Afulani PA, Diamond-Smith N, Phillips B, Singhal S, Sudhinaraset M. Validation of the person-centered maternity care scale in India. Reprod Health. 2018;15(1):147. 10.1186/s12978-018-0591-7 PMC6114501 30157877

[36] Kay ES, Rice WS, Crockett KB, Atkins GC, Batey DS, Turan B. Experienced HIV-Related Stigma in Health Care and Community Settings: Mediated Associations With Psychosocial and Health Outcomes. J Acquir Immune Defic Syndr 1999. 2018;77(3):257–63. 10.1097/QAI.0000000000001590 PMC5807196 29140873

[37] Nyblade L, Jain A, Benkirane M, Li L, Lohiniva AL, McLean R et al. A brief, standardized tool for measuring HIV-related stigma among health facility staff: results of field testing in China, Dominica, Egypt, Kenya, Puerto Rico and St. Christopher & Nevis. J Int AIDS Soc [Internet]. 2013;16(3Suppl 2). [cited 2019 Sep 24] Available from: https://www.ncbi.nlm.nih.gov/pmc/articles/PMC3833189/10.7448/IAS.16.3.18718 PMC3833189 24242266

[38] Neufeld SAS, Sikkema KJ, Lee RS, Kochman A, Hansen NB. The development and psychometric properties of the HIV and abuse related shame inventory (HARSI). AIDS Behav. 2012;16(4):1063–74. 10.1007/s10461-011-0086-9 PMC3638003 22065235

[39] Afulani PA, Aborigo RA, Nutor JJ, Okiring J, Kuwolamo I, Ogolla BA, et al. Self-reported provision of person-centred maternity care among providers in Kenya and Ghana: scale validation and examination of associated factors. BMJ Glob Health. 2021;6(12):e007415. 10.1136/bmjgh-2021-007415 PMC8638154 34853033

[40] Nyblade L, Addo NA, Atuahene K, Alsoufi N, Gyamera E, Jacinthe S et al. Results from a difference-in‐differences evaluation of health facility HIV and key population stigma‐reduction interventions in Ghana. J Int AIDS Soc [Internet]. 2020;23(4). [cited 2020 Oct 8] Available from: https://onlinelibrary.wiley.com/doi/abs/10.1002/jia2.25483 10.1002/jia2.25483 PMC7180216 32329153

[41] West CP, Dyrbye LN, Sloan JA, Shanafelt TD. Single item measures of emotional exhaustion and depersonalization are useful for assessing burnout in Medical Professionals. J Gen Intern Med. 2009;24(12):1318–21. 10.1007/s11606-009-1129-z PMC2787943 19802645

[42] Maslach C, Jackson S, Leiter M. Maslach Burnout Inventory Manual. 4th ed. Mind Garden, Inc.; 2016.

[43] Cichowitz C, Watt MH, Mmbaga BT. Childbirth experiences of women living with HIV: a neglected event in the prevention of mother-to-child transmission care continuum. AIDS. 2018;1. 10.1097/QAD.0000000000001860 PMC6029874 29762160

